# Impact of a VAP bundle in Belgian intensive care units

**DOI:** 10.1186/s13613-018-0412-8

**Published:** 2018-05-21

**Authors:** Laurent Jadot, Luc Huyghens, Annick De Jaeger, Marc Bourgeois, Dominique Biarent, Adeline Higuet, Koen de Decker, Margot Vander Laenen, Baudewijn Oosterlynck, Patrick Ferdinande, Pascal Reper, Serge Brimioulle, Sophie Van Cromphaut, Stéphane Clement De Clety, Thierry Sottiaux, Pierre Damas

**Affiliations:** 1 Service de Soins Intensifs Généraux, Domaine Universitaire du Sart-Tilman, Centre Hospitalier Universitaire, 4000 Liège, Belgium; 2 Dienst Intensieve Zorgen, VUB – Universitair Ziekenhuis Brussel, Campus Jette Laarbeeklaan 101, 1090 Brussels, Belgium; 3 Pediatrische Intensieve Zorgen, Universitair Ziekenhuis Gent, De Pintelaan 185, 9000 Ghent, Belgium; 4 Dienst Intensieve Zorgen, Algemeen Ziekenhuis Sint-Jan Brugge-Oostende, Ruddershove 10, 8000 Brugge, Belgium; 5 Service Soins Intensifs et Urgences, Hôpital Universitaire des Enfants Reine Fabiola, Avenue Crocq 15, 1020 Brussels, Belgium; 6 Urgentiegeneeskunde, Algemeen Ziekenhuis Sint-Maria, Ziekenhuislaan 100, 1500 Halle, Belgium; 7 Intensieve Zorgen, Universitair Ziekenhuis Onze Lieve Vrouw, Moorselbaan 164, 9300 Aalst, Belgium; 8 Anesthesiologie – Kritieke Diensten, Ziekenhuis Oost-Limburg, Campus Sint-Jan, Schiepse Bos 6, 3600 Genk, Belgium; 9 Intensieve Zorgen, Universitair Ziekenhuis Leuven, Herestraat 49, 3000 Louvain, Belgium; 10 Service de Soins Intensifs, Centre Hospitalier Universitaire Brugmann, Site Horta, Place Arthur Van Gehuchten 4, 1020 Brussels, Belgium; 11 Service de Soins Intensifs, Le Tilleriau, CHR Haute Senne, Chaussée de Braine 49, 7060 Soignies, Belgium; 12 Service de Soins Intensifs, Hôpital Erasme, Route de Lennik 808, 1070 Brussels, Belgium; 13 Intensieve Zorgen, UZA, Wilrijkstraat 10, 2650 Edegem, Belgium; 14 Service de Soins Intensifs et Urgences Pédiatriques, Cliniques Universitaires Saint-Luc, UCL, Avenue Hippocrate 10, 1200 Brussels, Belgium; 15 Soins Intensifs, Clinique Notre-Dame de Grâce, Chaussée de Nivelles, 212, 6041 Gosselies, Belgium

**Keywords:** VAP, VAP bundle, Belgian ICUs, VAP survey

## Abstract

**Background:**

In order to decrease the incidence of ventilator-associated pneumonia (VAP) in Belgium, a national campaign for implementing a VAP bundle involving assessment of sedation, cuff pressure control, oral care with chlorhexidine and semirecumbent position, was launched in 2011–2012. This report will document the impact of this campaign.

**Methods:**

On 1 day, once a year from 2010 till 2016, except in 2012, Belgian ICUs were questioned about their ventilated patients. For each of these, data about the application of the bundle and the possible treatment for VAP were recorded.

**Results:**

Between 36.6 and 54.8% of the 120 Belgian ICUs participated in the successive surveys. While the characteristics of ventilated patients remained similar throughout the years, the percentage of ventilated patients and especially the duration of ventilation significantly decreased before and after the national VAP bundle campaign. Ventilator care also profoundly changed: Controlling cuff pressure, head positioning above 30° were obtained in more than 90% of cases. Oral care was more frequently performed within a day, using more concentrated solutions of chlorhexidine. Subglottic suctioning also was used but in only 24.7% of the cases in the last years. Regarding the prevalence of VAP, it significantly decreased from 28% of ventilated patients in 2010 to 10.1% in 2016 (*p* ≤ 0.0001).

**Conclusion:**

Although a causal relationship cannot be inferred from these data, the successive surveys revealed a potential impact of the VAP bundle campaign on both the respiratory care of ventilated patients and the prevalence of VAP in Belgian ICUs encouraging them to follow the guidelines.

## Background

Ventilator-associated pneumonia (VAP) is among the most common type of intensive care unit (ICU)-acquired infection and is associated with significant morbidity and mortality [1]. In Europe, the incidence remains higher than in the USA despite the implementation of VAP bundles [2–4]. The need for the implementation of multimodal approach to decrease the incidence of VAP has been recently reemphasized by European guidelines [5] and especially by guidelines coming from the société française d’anesthésie-réanimation and the société de réanimation de langue française [6, 7]. Besides the use of selective digestive decontamination, these guidelines support the use of 6 procedures: avoiding intubation by the use of noninvasive ventilation, avoiding nasotracheal intubation, controlling cuff pressure, reducing the level of sedation, early enteral nutrition and subglottic suctioning.

In Belgium, after having observed high rate of VAP in ICUs from previous surveys, the federal service launched a promotional campaign to implement a national VAP bundle in 2011. This campaign involved several meetings in Brussels (attended by representatives from most Belgian ICUs) where the Belgian VAP bundle was explained and promoted. This campaign was followed by a prospective collect of all the VAP bundle data during 11 months in 2012 from voluntary participating Belgian ICUs. This collect was performed by the federal service. The national VAP bundle involved 4 items: a protocol with daily assessment of sedation, a semirecumbent position of at least 30°, the control of cuff pressure between 20 and 30 cm of H2O and the oral care with chlorhexidine. In addition, the use of subglottic suctioning was encouraged. Before and after this campaign, the college of physicians for intensive care, which also relies on the federal public service for health, food chain safety and environment, has performed surveys to evaluate the prevalence of VAP in Belgian ICUs. The present paper describes the results of the successive surveys and will examine the impact the campaign could have on the compliance of medical teams for implementing the bundle and on the prevalence of VAP. Data from the 2012 national collection study have been already published [8].

## Methods

Once a year, from 2010 till 2016, except in 2012, all the 120 adult ICUs in Belgium received an invitation to participate in 1-day survey performed by the college of physicians for intensive care about ventilated patients and the occurrence of VAP. ICUs were asked about their number of beds, their occupancy, the number of ventilated patients. Ventilated patient characteristics included age, sex, primary reason for ICU admission, comorbidities, date of admission to the hospital and to the ICU, date of intubation and cause of ventilation. Regarding ventilation care, the way of intubation (nasal, oral, tracheostomy),the type of cuff (polyvinyl, polyurethane), the current cuff pressure, the type of suctioning system (opened or closed), the current head positioning, the moistening system (heat and moisture exchangers, active devices) and the use of a subglottic suctioning system were recorded for each patient. Regarding the oral care, the type of disinfection (chlorhexidine, polyvidone iodine, other), the rate of disinfection, the use of dental brushing and the type of nutrition tubing (nasogastric, orogastric, postpyloric tube, gastrostomy or jejunostomy) were also recorded for each ventilated patients. If a patient was treated for a VAP, the bacteriological results were asked for and the severity of the infection according to the grade of sepsis was recorded. No follow-up of patients was obtained.

VAP diagnosis was based on new infiltrate on chest X-ray with either fever above 38° or less than 35° or leukocytosis above 10,000 white blood cells/mm^3^ and either occurrence of purulent tracheal secretions or decrease in PaO2/FiO2. After each survey, all the ICUs, having or not participated in the survey, received a report describing the results and were encouraged to continue to implement the VAP bundle.

### Statistical analysis

Quantitative data were summarized as median and interquartile (IQR) values or as mean and SD when normally distributed. Comparisons were made by the Kruskal–Wallis or Student’s *t* test as appropriate for continuous variables and by Chi-square or Fisher’s exact test for categorical variables. All tests were two-sided, and statistical significance was set at *p* less than 0.05.

## Results

**Fig. 1:**
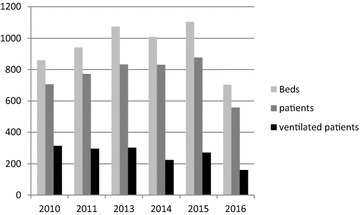
Evolution of the number of ICU beds, patients and ventilated patients in participating ICUs

More than 60 adult ICUs participated in the surveys, except in 2016 when there were only 44. Considering that in Belgium there are 120 acute hospitals, these figures correspond to 36.6–54.8% of them. Figure 1 gives the evolution of the number of ICU beds belonging to participating ICUs, the number of patients and among them, the number of ventilated patients. As can be inferred from Fig. 1, the percentage of bed occupancy remained stable, between 75 and 80%, but the percentage of ventilated patients decreased significantly from 44.8% in 2010 to 28.7% in 2016 (*p *< 0.05). Another impressive difference between the surveys was the decrease in the duration of ventilation from the ICU admission till the day of the survey: The median was 10.5 and 13 days before the campaign, then 7, 5, 5 and 6 days after the campaign (*p* < 0.001) (Table 1).

**Table 1:** Characteristics of ventilated patients

	Characteristics of patients						
	2010	2011	2013	2014	2015	2016	*p* value
Number	129	293	288	265	271	158	
Age median (IQR)	67 (54–78)	65 (54–75)	66 (54–75)	67 (57–76)	64 (54–76)	64 (50–71)	0.8412
Sex (male)	88 (68.8%)	183 (62.5%)	183 (63.5%)	162 (61.1%)	170 (62.7%)	102 (64.6%)	0.8326
Pre-ICU hosp. stay	0 (0–4)	0 (0–2)	0 (0–2)	1 (0–5)	0 (0–2)	0 (0–2)	0.3229
ICU stay median	10 (4–19)	13 (7–21)	8 (3–18)	7 (2–16)	7 (2–16)	8 (2–19)	< 0.0001
Duration of ventilation median	10.5 (4–17)	13 (7–21)	7 (3–16)	5 (2–14)	5 (1–15)	6 (2–14)	< 0.0001
*Type of patients*							0.0655
Medical	71 (55.8%)	156 (53.2%)	155 (53.2%)	138 (52.1%)	153 (56.5%)	94 (59.4%)	
Scheduled surgery	12 (9.3%)	51 (17.4%)	57 (19.8%)	52 (19.6%)	45 (16.6%)	18 (11.4%)	
Nonscheduled surgery	32 (24.8%)	51 (17.4%)	48 (16.7%)	59 (22.3%)	42 (15.5%)	30 (19%)	
Trauma	13 (10.1%)	35 (16.1%)	28 (9.7%)	16 (6%)	31 (11.4%)	16 (10.1%)	
*Underlying disease*							0.2724
Smoker	44 (34.1%)	102 (47%)	103 (35.7%	75 (28.3%)	81 (29.9%)	51 (32.3%)	
Asthma	7 (5.4%)	13 (4.4%)	5 (1.7%)	19 (7.2%)	9(3.3%)	5 (3.2%)	
COPD	36 (27.9%)	83 (28.3%)	89 (30.9%)	63 (23.8%)	64 (23.6%)	38 (24%)	
Solid cancer	4 (3.1%)	36 (12.3%)	28 (9.7%)	28 (10.6%)	20 (7.4%)	12 (7.6%)	
Hematological cancer	7 (5.4%)	7 (2.4%)	10 (3.5%)	4 (1.5%)	9 (3.3%)	4 (2.5%)	
Immunosuppression	12 (9.3%)	25 (8.5%)	20 (6.9%)	19 (7.2%)	16 (5.9%)	6 (3.8%)	
Corticotherapy	9 (7%)	37 (12.6%)	28 (9.7%)	39 (14.7%)	27 (10%)	15 (9.5%)	
Diabetes	13 (10.1%)	38 (13%)	42 (14.6%)	34 (12.8%)	34 (12.5%)	17 (10.8%)	
Other	NA	NA	NA	26 (9.8%)	26 (9.6%)	75 (47.4%)	
*Cause of ventilation*							0.0012
Hypoxia	50 (38.8%)	79 (27%)	95 (33%)	83 (31.3%)	80 (29.5%)	55 (34.8%)	
Hypercapnia	7 (5.4%)	39 (13.3%)	24 (8.3%)	19 (7.2%)	11 (4.1%)	16 (10.1%)	
Central nervous system	24 (18.6%)	47 (16%)	40 (13.9%)	32 (12.1%)	45 (16.6%)	19 (12%)	
Peripheral nervous system	0 (0%)	1 (0.3%)	2 (0.7%)	4 (1.5%)	5 (1.8%)	2 (1.3%)	
Trauma	7 (5.4%)	14 (4.8%)	12 (4.2%)	9 (3.4%)	14 (5.2%)	10 (6.3%)	
Circulatory problem	17 (13.2%)	38 (13%)	40 (17.4%)	40 (15.1%)	43 (15.9%)	17 (10.8%)	
Postoperative	17 (13.2%)	30 (10.2%)	59 (20.5%)	67 (25.2%)	63 (23.2%)	25 (15.8%)	
Other	7 (5.4%)	13 (1.1%)	16 (5.6%)	11 (4.1%)	10 (3.7%)	14 (8.9%)	

*IQR* Interquartile range, *ICU* intensive care unit, *COPD* chronic obstruction pulmonary disease, *NA* not available

The other characteristics of ventilated patients remained quite the same throughout the years of the surveys as shown in Table 1: age, sex, pre-ICU hospitalization stays, types of patients, underlying diseases, none of these characteristics differ between years of survey. However, regarding the causes of ventilation, the differences reached the statistical significance (*p* = 0.0012).

Regarding ventilatory care, it profoundly changed before and after the national VAP bundle campaign: As shown in Table 2, the cuff pressure measurement, which was not performed in 27% of ventilated patients in 2011, was obtained in more than 90% of cases in 2015. Head positioning above 30° was seen in 2010 only in 54% of ventilated patients, and it was systematically observed in more than 90% of the patients after the campaign except in 2016, when it decreased to 88.6%. Subglottic suctioning also significantly increased, and it was however used in only 24.7% of the cases in 2016. In the same way, oral care was more frequently performed, using more concentrated solutions of chlorhexidine as shown in Table 3. Other oral disinfectants were less often used (from 15.2% in 2010 to 6.3% in 2016), polyvidone iodine solutions remaining at a level of 34.8% in 2016. Dental brushing which was not performed in 25% of the ventilated patients in 2011 was still not done in 17.1% of the patients in 2016.

**Table 2:** Ventilatory care

	2010	2011	2013	2014	2015	2016	*p* value
Previous NIV	25 (19.4%)	50 (17.1%)	52 (18%)	46 (17.4%)	44 (16.2%)	33 (20.9%)	0.8685
*Artificial airway*							0.0663
Oral intubation	95 (75.4%)	244 (83.3%)	234 (81.2%)	222 (83.8%)	226 (83.4%)	132 (83.5%)	
Nasal intubation	0	2 (0.7%)	4 (1.4%)	4 (1.6%)	6 (2.2%)	2 (1.3%)	
Tracheostomy	34 (26.4%)	47 (16%)	50 (17.4%)	39 (14.7%)	39 (14.4%)	24 (15.2%)	
*Cuff*							0.0318
Polyvinyl	74 (64.3%)	166 (56.6%)	170 (59%)	143 (54%)	158 (58.3%)	110 (69.6%)	
Polyurethane	41 (36.7%)	127 (43.3%)	118 (41%)	122 (46%)	113 (41.7%)	48 (30.4%)	
*Cuff pressure*							< 0.0001
Not measured	9 (7%)	72 (24.6%)	2 (0.7%)	10 (3.8%)	4 (1.5%)	6 (3.8%)	
< 20 cm H_2_O	25 (19.4%)	18 (6.1%)	9 (3.1%)	9 (3.4%)	2 (0.7%)	7 (4.4%)	
20–30 cm H_2_O	91 (70.5%)	196 (66.9%)	263 (91%)	228 (86%)	251 (92.6%)	137 (86.7%)	
> 30 cm H_2_O	4 (3.1%)	6 (2%)	10 (3.5%)	15 (5.7%)	10 (3.7%)	5 (3.2%)	
Not inflated	0	1 (0.3%)	4 (1.4%)	3 (1.1%)	(1.5%)	3 (1.9%)	
*Suctioning system*							< 0.0001
Opened	76 (62.8%)	236 (80.5%)	204 (70.8%)	171 (64.5%)	205 (75.6%)	120 (79.7%)	
Closed	45 (37.2%)	57 (19.5%)	84 (29.2%)	94 (35.5%)	66 (24.4%)	38 (20.3%)	
*Subglottic suctioning*							< 0.0001
Yes	7 (5.8%)	67 (22.9%)	94 (32.6%)	60 (22.6%)	70 (25.8%)	39 (24.7%)	
No	114 (94.2%)	226 (77.1%)	194 (69.2%)	205 (77.4%)	201 (74.2%)	119 (75.3%)	
*Head position*							< 0.0001
< 30°	55 (44.4%)	43 (14.7%)	21 (7.3%)	13 (4.9%)	25 (9.2%)	18 (11.4%)	
> 30°	67 (54.0%)	249 (85%)	262 (91%)	249 (94%)	245 (90.4%)	140 (88.6%)	
Prone position	2 (1.6%)	1 (0.3%)	5 (1.7%)	3 (1.1%)	1 (3.7%)	0 (0%)	

*NIV* Noninvasive ventilation

**Table 3:** Oral care

Oral disinfection	2010	2011	2013	2014	2015	2016	*p* value
Water	1 (1%)	6 (2%)	1 (0.3%)	0 (0%)	0 (0%)	0 (0%)	
CHXD 0.2%	48 (38.4%)	126 (43%)	108 (37.5%)	132 (49.8%)	90 (33.2%)	79 (50%)	< 0.0001
CHXD 0.5%	13 (10.4%)	5 (1.7%)	11 (3.8%)	7 (2.6%)	4 (1.5%)	5 (3.2%)	
CHXD 1%	0 (0%)	2 (0.7%)	26 (9%)	7 (2.6%)	16 (5.9%)	3 (1.9%)	
CHXD 2%	0 (0%)	0 (0%)	10 (3.5%)	9 (3.4%)	15 (5.5%)	7 (4.4%)	
Polyvidone iodine	44 (35.2%)	119 (40.6%)	102 (35.4%)	93 (35.1%)	124 (45.8%)	55 (34.8%)	
Other	19 (15.2%)	35 (11.9%)	30 (10.4%)	17 (6.4%)	22 (8.11%)	10 (6.3%)	
*Rate of disinfection*							
1/day	2 (1.6%)	31 (10.6%)	3 (1%)	9 (3.4%)	2 (0.7%)	0 (0%)	< 0.0001
2/day	24 (19.2%)	41 (14%)	24 (8.3%)	27 (10.2%)	22 (8.1%)	8 (5.1%)	
3/day	36 (28.8%)	117 (39.9%)	158 (54.9%)	136 (51.3%)	116 (42.8%)	75 (47.4%)	
> 3/day	63 (50.4%)	104 (35.5%)	103 (35.8%)	93 (35.1%)	131 (48.3%)	75 (47.4%)	
*Dental brushing*							
0/day	NK	63 (21.5%)	51 (18.7%)	32 (12.9%)	36 (13.3%)	27 (17.1%)	0.0235
1/day	NK	99 (33.8%)	103 (35.8%)	88 (33.3%)	83 (30.6%)	63 (39.9%)	
2/day	NK	70 (23.9%)	63 (21.9%)	81 (38.6%)	84 (31%)	51 (32.3%)	
2/day	NK	46 (15.7%)	47 (16.3%)	45 (17%)	53 (19.6%)	11 (7%)	
Not applicable	NK	15 (5.1%)	25 (8.7%)	19 (7.2%)	15 (5.5%)	6 (3.8%)	

*CHXD* Chlorhexidine, *NK*: not known

**Fig. 2:**
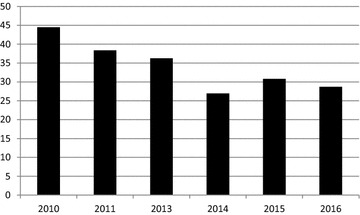
Percentage of ventilated patients treated for VAP the day of the survey

Regarding the prevalence of VAP, it significantly decreased from 28% of ventilated patients in 2010 to 10.1% in 2016 as shown in Fig. 2 (*p*  ≤ 0.0001). Interestingly, the associated bacteremia also decreased in absolute terms (from 7 to 2) but not relatively (from 8.9 to 12.5% of the corresponding VAP, *p* = 0.9625).

## Discussion

This paper reports on the implementation of a VAP bundle in Belgium. It was indeed expected in 2012 by the federal authorities to at least halve the VAP incidence encountered in Belgium. According to Fig. 2, this seems to have been reached and even exceeded, since the prevalence of VAP, as estimated by the surveys, was as high as 28% of the ventilated patients in 2010, reached 11.3% in 2014 and was maintained at this level for the next 2 years. It does not correspond to prevalence levels reported in the USA, but it corresponds to the best levels reported in European centers where VAP bundles are implemented [3, 4].

There is no clear consensus about what a VAP bundle should be [9, 10]. The first VAP bundle from the IHI includes elements which were not directly linked to the management of the airway (prevention of both thromboembolism and digestive hemorrhage) [11]. European experts proposed in 2010 only two procedures in addition to general measures such as hand hygiene, staff education and nonventilatory circuit change: control of sedation with protocol of weaning and oral care with chlorhexidine [12]. The Belgian VAP bundle [8] includes these two procedures into 4 components (sedation protocol, control of cuff pressure, oral disinfection, head of bed elevation) which were also parts of bundles recently published with reported efficacy [3, 4]. It also promotes a fifth procedure: subglottic suctioning which seems to have become the most useful procedure for the prevention of VAP [13–15].

The most striking difference between surveys appeared to be the percentage of ventilated patients and the duration of ventilation from the ICU admission till the day of the survey. Although this latter parameter was not the true duration of ventilation, it reflects a reality which means that from year to year, at one point in the year, fewer patients were ventilated and especially for less time in Belgian ICUs. Of course, this reduction of duration of ventilation reduces the risk for occurrence of VAP, estimated by these 1-day prevalence surveys. It would have been worth correlating this reduction of ventilation to a change in sedation procedures, which, unfortunately, were not directly assessed by the surveys. It may however be inferred that the campaign had an impact because of the clear difference between the duration before and after it.

After the campaign, the compliance with head of bed elevation and control of cuff pressure increased significantly and exceeded 90%. Oral disinfection also improved. The type of antiseptic varied largely, but the rate of application increased: It was done at least 3 times a day in 79.2% in 2010 and in 94.8% in 2016 (*p* < 0.0001). Chlorhexidine concentration of 1 or 2% was more often used after the campaign, but the low concentration of 0.2% was still used in 50% in 2016. The type and the concentration of antiseptic remain a matter of debate. Chlorhexidine has been shown as effective with concentration as high as 2% in a well-conducted multicenter study in the Netherlands by Koeman in 2006 [16]. Chlorhexidine has been, however, reported to be sometimes not well tolerated by the patients [17]. More worrying is that an increase in mortality in patients receiving chlorhexidine as part of oral disinfection was recently reported by Komplas [18] although this agent is used worldwide [19]. The merit of polyvidone iodine in oral disinfection is supported by very few studies [19], although it remains used in Belgium in one-third of the patients. Regarding dental brushing, it is surprising that as many as 17.1% of patients did not benefit from this care in 2016. They were already 21.5% in 2010, though dental brushing is the only way to eliminate or reduce the dental plaque which can contain a lot of pathogenic bacteria [20]. However, even if dental brushing has been shown to reduce the rate of pneumonia in postoperative patients [21], this was curiously not yet confirmed for VAP in ventilated ICU patients [22]. Subglottic suctioning, which was promoted, remains used in Belgium in a minority of patients. This could be due to the cost of the endotracheal tube which is on average 10 times higher than conventional tubes in Belgium. However, this procedure should still be encouraged because of its efficacy reported as high as 50% reduction of VAP incidence [15].

Thus, the VAP bundle was rather correctly followed and VAP incidence decreased. Was there a clear causal relationship between these two facts? These surveys cannot ascertain that statement, but they were carried out to control the expected impact on VAP prevalence. But the question remains of the reality of this impact, especially because the effect was seen only in 2014, 2015 and 2016, while the campaign was conducted in 2012. All the improvements seen in the application of VAP bundle were, however, already obtained in 2013. Why did the reduction in VAP prevalence not occur in 2013? In fact, this question is not anecdotic, because there may have been a change in the way the VAP was diagnosed and the reduction seen in 2013 onward could be due to this change. As said by Komplas, “an apparent decrease in VAP rate could be achieved by maximally exploiting the subjectivity and inconstancies of VAP definitions” [23]. In our opinion, it was not the case, because the way of diagnosing VAP remains the same, based on radiological findings, the occurrence of fever, change in leukocytosis and bacteriological results. The surveys were answered on a voluntary basis, and there were no reasons to minimize the VAP prevalence at any time. Most medical teams were the same during the surveys. An indirect evidence of the reality in the reduction of VAP prevalence was the corresponding decrease in associated bacteremia, the rate relating to the number of VAP being stable. That means that the same type of infections was taken into account during the surveys. However, Komplas’ concern regarding the data manipulation, even if unconscious, may still be real.

This report is not a true study such as the one recently published about the Spanish experience [24]. It is only a presentation of several surveys supporting the implementation of a VAP bundle in ICU ventilated patients. It gives figures from a large number of ICUs, allowing to describe the average activity of intensive care in Belgium and to encourage Belgian teams to prevent the occurrence of VAP by all valuable means. It is indeed interesting to observe the steady decline in number of ventilated patients in Belgian ICUs and duration of ventilation over time. Data quality may be, however, questioned because data could not be controlled, but remained consistent over time.

## Conclusion

The occurrence of VAP was a real issue in 2010 in Belgium. The efforts made by the medical and nurses teams of the different ICUs seem to have successfully contributed to the decrease in VAP prevalence which has now reached a low plateau for several years. However, there could still be room for further improvement.

## Abbreviations

VAP: ventilator-associated pneumonia
ICU: intensive care unit
IQR: interquartile range
SD: standard deviation
COPD: chronic obstruction pulmonary disease
NA: not available
NIV: noninvasive ventilation
CHXD: chlorhexidine
NK: not known
